# When and Why Placebo-Prescribing Is Acceptable and Unacceptable: A Focus Group Study of Patients' Views

**DOI:** 10.1371/journal.pone.0101822

**Published:** 2014-07-09

**Authors:** Felicity L. Bishop, Lizzi Aizlewood, Alison E. M. Adams

**Affiliations:** 1 Centre for Applications of Health Psychology, University of Southampton, Southampton, Hampshire, United Kingdom; 2 Department of Biology, Northern Arizona University, Flagstaff, Arizona, United States of America; The University of New South Wales, Australia

## Abstract

**Background:**

Surveys of doctors suggest that they use placebos and placebo effects clinically to help patients. However, patients' views are not well-understood. We aimed to identify when and why placebo-prescribing in primary care might be acceptable and unacceptable to patients.

**Methods:**

A purposive diverse sample of 58 English-speaking adults (18 men; aged 19–80 years) participated in 11 focus groups. Vignettes describing doctors prescribing placebos in primary care were used to initiate discussions. Data were analyzed inductively.

**Results:**

Participants discussed diverse harms and benefits of placebo-prescribing for individual patients, carers, healthcare providers, and society. Two perspectives on placebo-prescribing were identified. First, the “consequentialist” perspective focused on the potential for beneficial outcomes of placebo-prescribing. Here, some participants thought placebos are beneficial and should be used clinically; they often invoked the power of the mind or mind-body interactions. Others saw placebos as ineffective and therefore a waste of time and money. Second, the “respecting autonomy” perspective emphasized the harms caused by the deceptive processes thought necessary for placebo-prescribing. Here, participants judged placebo-prescribing unacceptable because placebo-prescribers deceive patients, thus a doctor who prescribes placebos cannot be trusted and patients' autonomy is compromised. They also saw placebo-responders as gullible, which deterred them from trying placebos themselves. Overall, the word “placebo” was often thought to imply “ineffective”; some participants suggested alternative carefully chosen language that could enable doctors to prescribe placebos without directly lying to patients.

**Conclusions:**

Negative views of placebos derive from beliefs that placebos do not work and/or that they require deception by the doctor. Positive views are pragmatic in that if placebos work then any associated processes (e.g. mechanisms, deception) are deemed unimportant. Public education about placebos and their effects is warranted and research to identify optimal ways of harnessing placebo effects in clinical practice is needed.

## Introduction

Placebo effects can be broadly defined as changes in a person's health status that result from the meaning and hope the person attributes to a procedure or event in a health care setting.[1], [2] These effects can be substantial[3]–[6] and are mediated by and involve measurable changes in neurological, immune, and endocrine systems.[7], [8] In clinical research, placebo effects must be controlled for (using, for example, placebo pills or sham surgery) but are typically not thought inherently interesting.[9] In clinical practice, placebo effects may have great relevance and potential to be harnessed for patient benefit[10], [11] and there are many ways doctors might attempt to do this. One approach is to enhance patients' expectations and communicate empathically, for example when managing pain in primary care[12], [13] or when delivering acupuncture.[14] This paper focuses on another, more controversial, approach to eliciting placebo effects: prescribing placebo-like substances such as sugar pills, “tonics” or low dose vitamins in primary care.

The actual contents of placebos are rarely reported even in clinical trials.[15] Recent surveys of clinical practice have distinguished between pure or inactive placebos (*e.g*. saline injections, sugar pills) and impure or active placebos (*e.g*. nutritional supplements in the absence of established deficiency, antibiotics for a viral infection).[16]–[18] The concept of impure placebos is however difficult to apply in practice as, for example, it is not always certain when prescribing antibiotics whether the patient has a bacterial or viral infection. Furthermore, doctors, patients, and researchers probably hold different definitions of placebos and may differ in whether they characterize particular interventions as being “real” treatments or placebos. [19] Overall, there remains little consensus regarding the definition of placebos and this probably contributes to wildly different estimates of the prevalence of placebo-prescribing in clinical practice. A recent systematic review found that estimates of the lifetime prevalence of prescribing pure placebos among doctors ranged from 17% to 80%[20] and it seems that doctors prescribe impure placebos more frequently than pure placebos.[20], [21]

For this study, we focused on scenarios which we believed most doctors and researchers would agree involve prescribing placebos in an attempt to elicit a beneficial placebo effect in patients. In such scenarios ethical issues abound, in particular the dilemma between principles of beneficence (prescribing placebos could benefit patients…) and respect for persons (…but prescribing pure placebos would require deceiving patients).[22]–[25] However, recent research confirms earlier suggestions that this dilemma might have shaky foundations: it might be possible to elicit clinically meaningful placebo effects in depression and irritable bowel syndrome by prescribing pure placebos openly without deceiving patients.[26], [27] If these initial findings are substantiated then this could encourage increased deliberate use of placebos in clinical practice to elicit placebo effects. What might patients make of this?

Very little is known about patients' views on placebos. Most studies have used quantitative methods[28]–[34] which cannot offer an in-depth understanding of patients' perspectives; qualitative studies have interviewed participants in clinical trials or experiments[35]–[38] but these settings are very different from clinical practice. We therefore conducted a focus group study to explore how members of the general public discuss placebo-prescribing in UK clinical practice. We used qualitative methods to discover inductively the issues that members of the public consider important. Our results identify when and most importantly *why* placebo-prescribing might be acceptable and unacceptable to patients in UK primary care.

## Methods

### Design

We chose to use focus groups to elicit lay people's views on placebo-prescribing in clinical practice because it is a potentially contentious topic and we know little about the issues that lay people deem relevant. Group settings encourage participants to both present and react to diverse opinions, which can help them to articulate and justify their own views. Unlike one-to-one interviews, focus groups thus allow researchers to observe the negotiation and formation of attitudes and norms within a group setting and to gain insight into the socially- and culturally-embedded reasoning processes that contribute to participants' judgments.[39], [40] To facilitate discussion of what could be quite an abstract and unfamiliar topic, we used short vignettes describing concrete examples of different ways in which doctors might prescribe placebos. Given that debates in this area have focused on the ethics of deception, we devised one vignette describing deceptive placebo-prescribing and one describing open-label placebo-prescribing. We also included vignettes about two contrasting illnesses (common cold and cancer), as we wanted to encourage participants to think broadly about different situations in which placebos might be used. Furthermore, as placebos are typically associated with clinical trials, we also included one scenario that described the use of placebos in a clinical trial. All of the vignettes described a doctor prescribing a placebo (rather than, for example, eliciting placebo responses by communicating empathically when prescribing an evidence-based condition-specific treatment). Together these vignettes provided the necessary basis for discussion but did not constrain the groups: participants were encouraged to introduce additional scenarios themselves and to adapt the vignettes in order to explore placebo-prescribing more fully.

#### Vignette 1: Clinical Practice with Deception

James is a healthy young man who goes to see Doctor Smith because he has a headache, a sore throat, and a runny nose. He feels pretty rotten. Doctor Smith examines him, and decides that James has a cold. Doctor Smith knows there is nothing seriously wrong with James, and knows that there is no medicine that will cure his cold. James will get better in his own time, as his body fights the cold virus. Doctor Smith gives James a prescription for a tonic anyway, and tells James that the tonic will help. James takes the tonic and feels better.

#### Vignette 2: Clinical Practice without Deception

Jake is another healthy young man who goes to see his doctor, Doctor Brown, because he has a headache, a sore throat, and a runny nose. He feels pretty rotten too. Doctor Brown examines him, and decides that Jake has a cold. Doctor Brown knows there is nothing seriously wrong with Jake, and knows that there is no medicine that will cure his cold. Jake will get better in his own time, as his body fights the cold virus. Doctor Brown explains all this to Jake. He then offers to give him a prescription for a tonic, saying that the tonic has no active medicine in it but might help to make him feel better anyway. Doctor Brown says that the tonic can be effective even without any active medicine, just because Jake wants it to work. Jake takes the tonic and feels better.

#### Vignette 3: Clinical Practice for Terminal Illness

Annie has advanced cancer. She has already had surgery, radiation treatment and chemotherapy. These treatments have given her a bit more time with her family. Sadly though, there is no cure for the particular type of cancer that Annie has. Dr Jones is Annie's doctor. Dr Jones wants to make sure that Annie is as comfortable as possible. Annie is still feeling hopeful, and Dr Jones does not want to dash her hopes. So, Dr Jones gives Annie some vitamin pills. The pills will not cure Annie's cancer. Dr Jones tells Annie that the pills are a type of treatment for people who have cancer.

#### Vignette 4: Clinical Trials

Medical researchers think that they might have come up with a new medicine for the common cold. They don't know if the new medicine will work or not, so to do a proper scientific test they have to compare their new medicine to a placebo medicine. People who have a cold agree to be in the clinical trial. They are randomly assigned (based on a coin toss) to get either the new medicine or the placebo medicine. The new medicine contains drugs that might help cold symptoms get better. The placebo medicine looks exactly like the new medicine but it is completely inert. It does not contain any active drugs. No-one knows which medicine each patient has had until the end of the trial. John agrees to take part in the trial, and his cold symptoms do get better. At the end of the trial, it turns out that he had the placebo medicine.

### Ethics Approval

Ethical approval was granted by the host institutions (University of Southampton and Totton College; ERGO ID584). Written informed consent was obtained using procedures approved by the ethics committee. To protect participants' anonymity, audio-recordings were destroyed following verification of the transcripts and completion of the data analysis. Transcripts were anonymised immediately (removing participants' names and other recognizable details *e.g*. towns, family members, clubs).

### Participants and Procedure

To capture the range of views held by members of the public we were guided by the concept of maximum variation sampling, an accepted approach to sampling in qualitative research. This approach is particularly appropriate when (as was true for the present study) there is little evidence to suggest in advance which contextual factors will shape the phenomenon of interest.[41] Therefore, we attempted to recruit a diverse sample of English-speaking adults including people who might reasonably be expected to hold different views on placebos. We aimed to include people with and without a scientific background; young, middle-aged and older adults; people from different socio-economic backgrounds; people with and without chronic illness; and people living in different regions of England (South, Midlands, South-West) and in urban (inner city, market town, suburban) and rural locations. Existing contacts in the community were used to invite pre-existing groups of people (helping to facilitate comfortable group discussions). Potential participants were informed of the study in writing and invited to take part; focus groups were organized in consultation with the volunteers at convenient community locations (*e.g*. a tea-room, village hall). 58 participants (18 men, 40 women aged 19–80 years) took part in 11 focus groups that successfully reflected the intended range of characteristics (see Table 1).

**Table 1:** Characteristics of Participants and Focus Groups.

Group	N	Age	Gender (n)		Chronic illness (n) a	Employment	English	Duration
		(range)	Male	Female			Region	(mins)
FG1	6	X	0	6	X	Psychology students	South	54
FG2	9	34–53	1	8	3	Mostly working	South West	62
FG3	7	54–75	3	4	6	Working, retired	South West	58
FG4	5	46–79	1	4	2	Retired	South West	51
FG5	5	49–80	2	3	1	Working, retired	South West	64
FG6	3	22–24	1	2	X	Health psychology students	South	31
FG7	4	21–23	2	2	X	Non- psychology students	South	28
FG8	6	19–71	3	3	X	Students, working, retired	Midlands	41
FG9	4	21–52	2	2	X	Students, working	Midlands	37
FG10	4	52–73	1	3	X	Retired	Midlands	25
FG11	5	24–49	2	3	X	Working	South	28

Note – X indicates data not collected.

a Self-reported.

Each focus group was audio-recorded and moderated (i.e., facilitated) by AA or LA, alone or with FB. AA and LA received training in qualitative research and were supervised by FB, who is an experienced qualitative researcher. The focus group moderator began by introducing herself (as a researcher interested in public views on placebos) before providing an overview of ethical issues (*e.g*. confidentiality among participants) and obtaining written informed consent. The moderator then initiated discussion among the participants by reading aloud one vignette and inviting comments. The moderator observed as the groups discussed their views, and put follow-up questions to the groups if necessary in order to ensure a full discussion and to encourage participants to explain their thinking in a non-judgmental manner. Broad and open questions were used for this purpose, for example: ‘How would you feel if the person had a more serious illness?’ ‘How do you think the placebo effect works?’ The moderator encouraged individual participants to speak if necessary to elicit full and detailed contributions and ensure that all participants had the opportunity to speak. Groups were brought to a close when all vignettes had been considered, views had been explored in depth and participants were satisfied that the important issues had been discussed.

### Data Analysis

Audio recordings were transcribed verbatim and analyzed inductively for themes.[42] This means that we did not use an a priori framework to code our data nor did we anticipate in advance specific themes or categories of talk. Instead, we analysed the data carefully and with open-minds in order to identify the issues and arguments that our participants themselves raised. The analytic process was iterative (*i.e*., moved forwards and backwards between different activities) and the six phases outlined by Braun and Clarke[42] were used to help develop themes that captured important patterns in the data in relation to participants' views on placebo-prescribing in primary care. First, recordings were listened to and transcripts were read repeatedly and slowly, meaning unit by meaning unit[43] and annotated with comments. A meaning unit is the smallest unit of speech that expresses a single meaning; often this corresponds with a phrase or a sentence. Second, inductive (data-driven) initial codes were developed and applied to all transcripts,[44] and collated in the form of a coding manual. An example of an inductive code is “doesn't matter how placebo works”, which was applied to excerpts such as “I wouldn't care whether the placebo worked, it doesn't matter particularly, as long as the medicine was a safe medicine. The point is do I get better? I don't really care how it happens, to be honest.” A coding manual lists all of the codes, defines each of them and provides an illustrative excerpt from the raw data. As such, it provides a transparent record of when each code should be used that forces researchers to be rigorous and specific in their coding, forms an important part of the ‘audit trail’ that documents the process of qualitative analysis and allows for the possibility of replication,[45] and can facilitate team-working during qualitative analysis. Third, higher level, more abstract potential themes were developed by merging and splicing the initial codes. This involved merging together two or more codes that stood for similar ideas or splicing them together to form a new potential theme that cut across the original codes. For example, a new, overarching, potential theme that was labeled “Placebos work by psychological mechanisms” was created by splicing together a number of codes that described different psychological mechanisms that participants thought might contribute to placebo effects, including: “feel better in your head”, “helps psychologically”, “mind over matter” and “positive thinking”. At this stage, themes were discussed by at least two researchers, to ensure that the analysis was not idiosyncratic, overly narrow, or excessively broad. Fourth, preliminary themes were reviewed for fit with the coded extracts, each transcript as a whole, and the entire data corpus. In other words, we checked that each preliminary theme summarized the ideas expressed in the excerpts coded under it; we also checked that when taken together the preliminary themes captured the ideas expressed in each transcript and across the entire set of transcripts. This ensured our themes captured the variation in our data, did not overlook any important issues or perspectives, and were an accurate interpretation of the data. We also reviewed how the preliminary themes related to each other. At this stage, some preliminary themes were re-conceptualized as *sub*themes of larger themes that encompassed related ideas; for example “placebos work by psychological mechanisms” and “role of personal interactions in placebo effects” were both conceptualized as subthemes of the theme “(How) do placebos work?” Fifth, the contents and name of each theme was finalized. Sixth, the analysis was written up and vivid and typical illustrative quotes were selected for inclusion in this report.

Procedures used to enhance the rigor of this qualitative analysis include: maintaining an audit trail (coding was documented in Atlas.ti which is a software designed to support qualitative analysis; a coding manual was produced; analytic memos and diagrams were used to record analytic ideas and decisions); deliberately searching for exceptions and contradictions to apparent patterns; having three researchers heavily involved in the analysis; sustained engagement with the data over time; presenting verbatim quotes and excerpts to illustrate themes and provide evidence grounding our interpretation in the data.[46]

## Results

In considering whether doctors might legitimately prescribe placebos in clinical practice, the focus groups discussed both the process and the likely outcomes of placebo-prescribing. When either the process or the outcomes were prioritized, clear judgments were made as to whether doctors should prescribe placebos. When attempts were made to balance concerns about both the process and the outcomes, talk about placebo-prescribing enacted a familiar ethical tension: should doctors tell the whole truth or should they lie to benefit their patients? More fundamentally, the notion that placebos can benefit patients was scrutinized by the groups.

### (How) Do Placebos Work?

Participants were unwilling to accept at face value the statements in our scenarios that implied placebos can benefit patients, and instead discussed this proposition (and possible explanations for it) in some detail. In doing so, participants drew on experiential knowledge gleaned from personal experiences of taking placebos, responding psychologically to conventional medicines, and using complementary and alternative medicines, particularly homeopathy. Participants also discussed more abstract “facts” about placebo effects, which were attributed to academic studies, radio and television programs; they discussed the role in illness and healing in general of psychological factors such as stress and thinking positively; and in some cases they turned to the focus group moderator to seek a more expert opinion on the veracity of placebo effects. Those who ultimately agreed that placebos could benefit patients expressed various models of how such effects might be achieved. Some models were no more than broad general notions that reverentially invoked the mysterious powers of the non-corporeal mind to influence the body, suggesting a degree of Cartesian duality, which led participants to struggle to comprehend how a psychological input could have a physiological effect. Other models focused on the brain, which seemed to allow a slightly clearer idea of how physiological effects could be produced by placebos containing “inactive” ingredients.

“I think these placebos are amazing to be honest these things because to be honest you know, people get better without ever having to take medicine just basically by the powers of their own mind.” (FG1)

“I’ve heard a lot about dopamine and taking placebos causing that to be released and I don't know what effect dopamine might have but there might be a mechanism where, by which (P: Makes you feel better) by which it actually has a physical effect. I suppose also there's like people don't completely understand the human body so there might be mechanisms that we don't even know about yet which cause people's mentality to change their physical condition.” (FG8)

Patients' individual characteristics were discussed as potentially influencing the effectiveness of placebos, with certain patients deemed more likely to respond well to a placebo than others. Some but not all of these judgments were somewhat derogatory: for example when talking about a character in the vignettes “I don't think he is very bright” (FG4) and “some patients need that psychological help” (FG6). When individual differences in placebo response were thought to be predictable, doctors who knew their patients well were seen as having an important role in ensuring that placebos are given to people who would benefit from them.

“If he was one of those patients that the doctor knew would expect something and would get better quicker because he's been given something, I don't see any harm in that.” (FG4)

The doctor-patient relationship was also seen as integral to the placebo effect itself. From this perspective, placebo effects were attributed to the doctor as a trusted authority figure providing reassurance and a positive message about recovery. This seemed to be a particularly accessible model of placebo effects, which participants could relate to. Indeed, some recounted their own experiences of reassurance from a trusted doctor leading to immediate improvements in wellbeing and symptoms.

“I don't know whether it is a placebo effect but sometimes you can go to the doctor feeling dreadful you know and you think oh there must be something seriously wrong, all these headaches, this pain and that pain and the other and you get there and you start to explain it and suddenly he sort of he'll go “oh, well that's because it's just your circulation which is a problem when you get older and that's this, is that” and by the end after 5 minutes you come out thinking I don't know why I ever went, I feel better already, really there's nothing wrong with me much.” (FG8)

### Consequentialist Perspectives: Effective Placebos are Acceptable in Clinical Practice

Placebos were only judged acceptable in clinical practice in certain circumstances, essential among which was the perception that placebos can benefit the patient. Some participants demonstrated a particularly strong consequentialist perspective, arguing that as long as placebos benefit the patient then they should be used in clinical practice. They prioritized potential benefits over other issues, including mechanisms of action and (dis)honesty.

“I don't think it matters as long as it works.” (FG1)

“The point is do I get better? I don't really care how it happens, to be honest.” (FG8)

In the context of serious illness, some participants also drew on a discourse of desperation to justify placebo-prescribing: provided the patient might benefit *and* certain other conditions were satisfied, they considered it would be “worth trying” placebos as a last resort. The necessary additional conditions for placebos to be acceptable here included the lack of any proven effective treatment, the placebo not causing harm, and the person being in pain or otherwise suffering.

“It depends how bad you're feeling – if you're feeling really bad, you might be at the point where it's just like I'll try anything. I just want to get better.” (FG1)

“If it'll help, it'll help and if there's no active medication that works, as long as it's not harmful it can only be helpful.” (FG6)

While effective placebos were acceptable to those taking a consequentialist perspective, the converse was also true: not surprisingly, when placebos were perceived as powerless or unnecessary, their use was deemed unacceptable. Some participants argued that because placebos lack active ingredients, they cannot “work” or have any effect on patients' health, which again was reminiscent of a Cartesian model in which a physical agent of change must be introduced to have a meaningful effect on symptoms. When placebos were deemed powerless, their use was seen as a waste of time and money that would be better spent on “real” treatment.

“I wouldn't - personally I wouldn't take it - because what is the point? It's a waste of money and a waste of resources. So no, I wouldn't do it.” (FG9)

“If the tonic hasn't really got any active ingredient, then it's not actually honest and I wouldn't want someone giving me something, you know, say it's going to make me better when actually it's of no use at all.” (FG5)

### Respecting Patients' Autonomy: Deceptive Placebo-Prescribing is Unacceptable in Clinical Practice

Almost all participants suggested that placebo-prescribing must be deceptive in order to be effective. When participants prioritized the deceptive nature of the prescribing process over other issues they judged placebo-prescribing unacceptable. Deceptive prescribing was seen as threatening a patient's right to self-determination and autonomy; doctors who prescribed placebos deceptively were seen as acting in an overly paternalistic patronizing way, making decisions for (instead of with) patients, not allowing patients the opportunity to fully comprehend their situation, and taking away patients' right to choose their own health care. Deceptive placebo-prescribing was condemned on these grounds across diverse scenarios by participants in all focus groups.

“the doctor must explain it and it would be very wrong to give somebody treatment that was inappropriate, very wrong. It's dishonest and it ruins the relationship between doctor and patient. It's much better to be honest about it.” (FG10)

“I would be furious, I have to say, if I did go to the GP and wanted – needed medication and I don't take medication at all unless I absolutely have to; if I do go and then find afterwards that it had been a placebo, without my permission, I would want to sue, I would be so angry.” (FG2)

Interestingly, there was one clear exemption from the requirement to respect a patient's autonomy and therefore not to prescribe placebos deceptively: when the recipient is a child. This is consistent with children's legal status in medical situations in which parental informed consent is necessary and child assent is desirable. Participants generally recognized that placebos are frequently given to children by their carers, citing examples of ‘magic plasters’ and ‘magic kisses’, and held the view that children are also more likely than adults to believe a placebo will make them feel better.

“Loads of times - like, especially when you're a kid, things like the magic kiss, where you have a fall or there is Calpol which is sugar, or there is like a certain like, my mother had us convinced that jelly tots were like going to make us better - regardless of the illness.” (FG6)

The only situation in which placebos were universally accepted was clinical trials. Although not the focus of this paper, it is relevant to note here that all of the focus groups were generally accepting of placebos in clinical trials, based on the assumption that patients would be fully informed and give consent.

“It's a trial which I think it's fine, I've got no objection about it being given in a trial at all because you know what you're signing up for.” (FG4)

### Fine Lines and Tightropes: Weighing Benefits and Harms

When participants considered both the process and outcomes of placebo-prescribing they attempted to weigh benefits and harms, which were broadly construed in relation to the individual patient, their family, the patient-doctor relationship, and society at large. In weighing benefits and harms some groups and individual participants oriented to a tension between (a) wanting doctors to be honest (to respect patients' autonomy) and (b) wanting doctors to do everything possible to help their patients. Participants recognized the potential for beneficial health outcomes from taking placebos and for harm from the deceptive prescribing process. Table 2 summarizes the diverse benefits and harms of placebo-prescribing that were discussed in the focus groups. The following excerpt from focus group 5 illustrates how these benefits and harms were debated in interaction.

**Table 2:** Benefits and Harms of Placebo-Prescribing as Discussed in Focus Groups.

Perceived Consequence of Placebo-Prescribing	Illustrative Quotation
**Benefits**	
Financial – for the NHS	Can I just go back to what you were saying, that that is an issue isn't it now, with the cost of the NHS; if a placebo is costing you a pound to give out and they're [patients are] paying £7.40, that could bring a lot of revenue for the NHS. (FG2)
Alleviate discomfort	‘‘I’d want them (an elderly person) to feel as comfortable as possible’ FG5
Benefits for carers	‘I have a parent who's crippled by arthritis and it's very severe, very nasty now, I think it's probably past the point of, very much, of being able to do very much about it; but I feel that by administering a placebo to one parent, the other parent probably benefits on the care side of things as well' FG2
Feeling cared for, supported	“Or is the doctor treating the needs of his patients? He knows that some people just say ‘right, I'll work through a cold’. whereas that patient needs a bit of caring, that little bit of showing - by giving them the tonic it's showing caring for his - and treating it like it is a real illness, so maybe there is an element of benefit in that. You know sometimes people want to be listened to or felt that someone's helping them, that could be a consideration, that the tonic, while it's not medically helping them, it's emotionally helping them.” (FG2)
General mood	I:OK. What effects do you think a placebo can have on people?
	MP: Just your general mood, if you believe you're being treated for something that's going to lift your spirits a little bit (FG11)
Symptom improvement	But she might believe it and then - be happy. Might relieve her pain when nothing else is available to do that. (FG7)
Avoids risk of addiction associated with pharmaceuticals	‘if the alternative to a placebo is something strong, like morphine, then I'd rather the placebo first, as morphine is addictive’. (FG5)
No adverse effects	You've also got that - the other advantage of - by giving them a pill they think they feel better, they're also not getting any of the side-effects. (FG2)
Maintain some hope	It's good to give her a little bit of hope. (FG11)
Reduce need for medicines	I just think GPs should never tell you, and just say take this and you're going to get better by the power of your own mind then you'll never have to take medicine again. (FG1)
**Harms**	
Financial – for the patient/NHS	MP: It's disingenuous in today's context where you're paying for a prescription and presumably if it's sugar water it doesn't come as expensive as a prescription charge does anyway, so he should tell him to go away and buy a tonic or whatever, you know, non-prescription drug because with the way that prices and costs of prescriptions are, if the chap finds out that he's being given something which is, you know, nothing really, he might be quite cross with the doctor even though he is feeling better later on. So -
	P: I hadn't thought of prescription charge, because it is a lot.
	P: What is a prescription charge?
	P: Seven pounds twenty.
Risk of missed/delayed diagnosis	“You've hit the nail on the head. It isn't about like - if it was a cold, it's a cold, it's a cold. It'll be a cold. But if it was like - if it was that he thought it were a cold and it turns out you had TB, then that's a completely different situation but as [other participant] says, that's where you kind of flit into malpractice.” (FG6)
If used as an alternative to proven treatment	“Well the only negative effect I'd say is when it's like a critical illness, where a fatality could be a consequence and there is a medicine that they could take which can battle the cancer and the doctor decides to do a placebo that has no effect.” (FG7)
False hope	“If someone's turned around and given you false hope and said these will help cure your cancer, then it's a different matter.” (FG11)
Patient feels disrespected	I would want the truth, I really don't like being fobbed off as if you're a nitwit. (FG3)
Threatens trust in doctor if deceit is discovered	‘I suppose that if he finds that they're only placebos or sugar pills, then you know, you lose trust in the doctor’. (FG11)
Put off going to doctor in future	“I think there's a danger though if he's found out that the doctor tricked him then he might not go to the doctor next time when it's something more serious.” (FG8)
Encourages over medicalization of minor illness	“But isn't it just going to lead to him going to the doctors again because he wants more tonic because he's got another cold?” (FG7)
Panders to patients' reliance on doctors	‘Somebody then becomes dependent on them and going to the doctor and getting one of those and that is what I mean about it being sinister because the whole society's expecting that something will make them better from whatever they've got all the time’ (FG5)
Unlikely to be effective	“But surely a placebo would only work with a handful of cases.” (FG8)
Removes patient choice	“What goes into my body; it's my body, it's up to me to decide, it's up to nobody else at all, you know, they are there to facilitate my healing, they are not in control of it.” (FG2)

P1: But placebos will only work if the doctor is dishonest. The question is - that if the, if it was just a common cold it will be cured in time, the body will cure itself; in that case dishonesty's justified I think, give out placebo, by all means and then, then make them believe it. I mean you certainly don't say that it doesn't contain any active ingredients. You don't tell them that.

P2: Well actually the doctor is saying that I haven't got anything that will cure you, but I do know that if you go home and have some whiskey and hot water with a bit of honey in it, it won't do you any good, but it will make you feel better.

P1: Yeah, yeah I'm not, not saying that placebos should be given, but I don't see any moral, any, any moral condemnation of placebos. I mean I don't think it's immoral of the doctor to give a placebo if he's convinced that the man has got nothing seriously wrong with him, but if he thinks that the man will be happy, then don't tell him that it's got no active ingredients, say here's this, try this, you. The doctor won't say this will cure you, all a doctor will say is try this you might get better.

P3: This may make the underlying problem worse cause then the person then becomes totally dependent on the doctor giving him something that isn't actually, is nothing more than a bit of TLC. (FG5)

In the context of minor illness (a common cold), a more moralistic discourse appeared in which placebo-prescribing was seen as harmful in that it wasted resources, represented unnecessary medicalization of minor illness and encouraged dependence on doctors. These participants saw the common cold as a minor self-resolving condition which patients were obliged to self-manage (*e.g*. through rest and home remedies) without seeking professional medical care.

“I don't think he really should've given him anything. He should tell him that he doesn't need anything, he's got a common cold and it will get better naturally.” (FG4)

Discussions of benefits and harms were particularly lively when participants considered the (deliberately provocative) vignette in which a patient with terminal cancer was prescribed vitamins as a placebo after having completed conventional treatment. While all but one group initially reacted very negatively to this scenario (suggesting possible consensus against placebo-prescribing in this situation), as discussions continued more diverse views were articulated. The language used in these discussions evoked dilemmas, with participants speaking of “tightropes” and “fine lines”. In terminal illness in particular, the dilemma was between the doctor being honest (but destroying hope) and providing benefit (but creating false hope). Uncertainty abounded in these discussions and consensus around a clear judgment on acceptability was rarely achieved, as illustrated by the following excerpt from focus group 7.

P3: Yeah, I agree with that but it's also kind of deception as well and it's not really fair. But as I said, it's with the best intentions. But it is morally weird.

P2: Yeah, I think you've said it already but what it comes down to is the pain on one side and just the truth –

P3: Yeah

P4: But you're assuming that the placebo's going to have an effect on the pain aren't you? Is a placebo just to do with pain, or is it more to do with your mood, your positive-ness and your general wellbeing?

P2: I think that would overcome the pain if she believes in the placebo, the pain level could be the same but like you said before with the positive, you know -

P4: How do you know? [interrupting]

P2: Mental attitude. You don't know, you can't know. But I'm sure, you know, they would have one-to-ones with their patients and if the feedback's a lot more positive then um I think I don't, well I do see a problem, but I see how it benefits. (FG7)

Some focus groups suggested that careful use of language might resolve the dilemma: if doctors are vague or tentative in how they describe placebos then they might be able to use them to elicit placebo effects without directly lying to patients. In other words, some participants suggested that careful wording (or “fudging” the truth) could be a way of avoiding “blatant” lies and thus rendering placebo-prescribing acceptable. This resolution was not satisfactory for all participants and there was considerable variation in the interpretation of particular words. For example, some considered “treatment” to imply cure while others considered it to imply merely that the substance might help the patient in some way. Other terminology and phrasings were also interpreted in diverse ways and the very term “placebo” was itself problematic. For some, “placebo” was to be avoided at all costs; for others, avoiding the word “placebo” constituted unacceptable deception. Many participants believed that using the term “placebo” would render placebos powerless.

“You can tell a person in so many words this is a placebo but if you don't say this is a placebo, they still hold on to a glimmer of hope. But when you say placebo people just stop believing it'll work at all, and they'll almost fight it. They'll want it to not work just to prove that they can't be influenced by the placebo.” (FG6)

## Discussion

### Principal Findings

Despite evidence indicating that placebos can be beneficial and lead to measurable physiological changes, there remains much professional uncertainty and contradictory views as to whether they should be used in the clinic. Surveys have also indicated a range of patient opinions on the acceptability of placebos, but to date there have been no studies examining the underlying beliefs that influence either positive or negative views. In this study, we were interested in determining when and why patients find placebos acceptable. We showed that uncertainty and contradictory views abound among the community. There was no overall consensus as to the acceptability of placebo-prescribing, for either minor self-limiting illness or terminal illness. Participants in our focus groups espoused two core perspectives. The *consequentialist* perspective was pragmatic and focused on the potential outcomes of placebo-prescribing: if placebos are beneficial, they should be used in the clinic. Such views were founded on beliefs that placebo-prescribing can have benefits for patients and ideas about how placebos might work often invoked the power of the mind or mind-body interactions. The *respecting autonomy* perspective emphasized the ethical harms caused by the deceptive processes thought necessary for placebo-prescribing: prescribing placebos in the clinic is unacceptable because it requires the doctor to deceive the patient. In their discussions, participants considered many potential harms and benefits including the implications of placebo-prescribing for patient and carer wellbeing, NHS and personal resources, doctor-patient relationships, medicalization of minor illness, and patient choice and the right to self-determination (for adults if not children). The word “placebo” was itself problematic as it was often thought to imply “ineffective”; some participants felt that doctors might be able to use alternative carefully chosen language to prescribe placebos such that beneficial effects could be realized without directly lying to patients.

### Strengths and Limitations

Using vignettes within focus groups allowed us to introduce a relatively unfamiliar topic in a way that prompted discussion and at times lively debate among members of the general public. Participants expressed and responded to diverse opinions, which allowed us to observe at first hand the arguments and counter-arguments that were brought to bear on the controversial topic of placebo-prescribing. Moreover, unlike surveys, focus groups gave participants the opportunity to discuss the topic with their peers in a social setting, to question our assumptions and to raise issues of importance to them that we – as researchers - might not have anticipated.

A limitation is that participants were recruited from a small sample of groups known either directly or indirectly to the researchers; therefore, the range of opinions expressed in the focus groups may be limited. The data we obtained, however, did reflect a wide range of opinions, and participants were recruited from a variety of ages, educational levels, social groups, and regions of England. A second limitation is that participants were asked to discuss hypothetical scenarios (the vignettes). If participants were offered placebos during a visit to the clinic they might respond differently. Finally, the vignettes focused on placebo-prescribing and did not describe the doctors' motivations. We wanted to explore patients' views about doctors deliberately attempting to elicit a beneficial placebo effect by prescribing a placebo (see Introduction). However, doctors have a wide range of reasons for prescribing placebo interventions in clinical practice, some of which emphasize benefits for the doctor more than the patient (e.g. to satisfy a patient's demand for a prescription; to feel able to “do something”; and to manage the clinical uncertainty that can be common in general practice).[47], [48] More qualitative studies are now needed to explore patients' views about these other types of placebo-prescribing that may already be common in practice.[47]

### Relation to Other Studies

Previous studies of patients' views regarding clinical applications of placebos have used surveys and/or questionnaires.[28]–[34] Most recently, Hull and colleagues surveyed adults in the USA to determine the acceptability of placebo treatments and found that most, but not all, respondents thought it was acceptable for doctors to use placebos under some circumstances.[34] However, although a significant number of patients (21.8% in the Hull *et al*. study) thought that placebos were not acceptable under any circumstances, the reasoning behind this view was not revealed in the surveys. Using focus groups in our study allowed us to obtain a more in-depth understanding of reasoning used to either support or oppose the use of placebos in the clinic. In particular, our findings suggest that people who find placebo-prescribing unacceptable do so because of beliefs that (i) placebos are ineffective, which leads to the view that placebos are a waste of time and money, and pander to dependency on doctors and medications; (ii) placebos require deception, which leads to the view that a doctor who prescribes placebos is not to be trusted, and that patients' involvement in their own healthcare is compromised; and (iii) patients that respond to placebos are gullible, foolish, or childish, leading to the view that they themselves would not want to be treated in this way. On the other hand, people who find placebo-prescribing acceptable seem to do so primarily because they believe placebos can be effective and they prioritize such patient benefit over other concerns.

### Implications

Our findings suggest that placebo-prescribing might be more acceptable to patients in some circumstances if they understood better that (i) placebos can trigger improvements in symptoms and other benefits for patients; (ii) deception may not be necessary for placebos to be effective (*e.g*., carefully worded explanations by doctors could obviate outright lying, more detailed explanations could lead to belief in placebos, and general consent forms could be used to give blanket permission to receive placebos); and (iii) the ability to respond to placebos takes advantage of biological mechanisms already in place in all humans (and therefore is not confined to the more ‘gullible’ sector of the population). Some patients might still deem placebo-prescribing unacceptable and this view would need to be respected by clinicians and researchers. Overall, however, it seems critical to find ways to educate patients about placebos and their effects.

### Unanswered Questions and Future Research

We do not know the extent to which our findings have captured the range of views held among the general public at large. Therefore, additional research could expand on these findings by running focus groups in other areas of the UK/elsewhere and with people from ethnic minorities not reached by this study; our findings could also be used as the basis to conduct a large-scale survey of beliefs and knowledge about placebos and placebo effects. In addition, more research is needed to test the efficacy of placebo-prescribing in the clinic and to identify the best ways for doctors to harness placebo effects, taking into account patients' diverse perspectives.

## Conclusions

Patients are uncertain and express diverse and contradictory beliefs about the acceptability of placebo-prescribing: some find it acceptable, for example when they prioritize possible benefits to patients; others find it unacceptable, for example when they believe placebo-prescribing must involve deception and thus violates a patients' right to self-determination and participation in healthcare. Public education about placebo effects is needed to correct misunderstandings. Additional research is warranted to identify optimal ways of harnessing placebo effects in clinical practice for patients with diverse views.
